# Preliminary Insights into the Impact of Dietary Starch on the Ciliate, *Neobalantidium coli*, in Captive Chimpanzees

**DOI:** 10.1371/journal.pone.0081374

**Published:** 2013-11-25

**Authors:** Kateřina Schovancová, Kateřina Pomajbíková, Petr Procházka, David Modrý, Petra Bolechová, Klára J. Petrželková

**Affiliations:** 1 Institute of Botany and Zoology, Masaryk University, Brno, Czech Republic; 2 Department of Pathology and Parasitology, University of Veterinary and Pharmaceutical Sciences, Brno, Czech Republic; 3 Institute of Vertebrate Biology, Academy of Sciences of the Czech Republic, Brno, Czech Republic; 4 Biology Center of the Academy of Sciences of the Czech Republic, Institute of Parasitology, České Budějovice, Czech Republic; 5 Central European Institute for Technology, Brno, Czech Republic; 6 Department of Husbandry and Ethology of Animals, CULS, Prague, Czech Republic; 7 Liberec Zoo, Liberec, Czech Republic; Université Catholique de Louvain, Belgium

## Abstract

Infections caused by the intestinal ciliate *Neobalantidium coli* are asymptomatic in most hosts. In humans and captive African great apes clinical infections occasionally occur, manifested mainly by dysentery; however, factors responsible for development of clinical balantidiasis have not been fully clarified. We studied the effect of dietary starch on the intensities of infection by *N. coli* in two groups of captive chimpanzees. Adult chimpanzees infected by *N. coli* from the Hodonín Zoo and from the Brno Zoo, Czech Republic, were fed with a high starch diet (HSD) (average 14.7% of starch) for 14 days, followed by a five-day transition period and subsequently with a period of low starch diet (LoSD) (average 0.1% of starch) for another 14 days. We collected fecal samples during the last seven days of HSD and LoSD and fixed them in 10% formalin. We quantified trophozoites of *N. coli* using the FLOTAC method. The numbers of *N. coli* trophozoites were higher during the HSD (mean ± SD: 49.0±134.7) than during the LoSD (3.5±6.8). A generalized linear mixed-effects model revealed significantly lower numbers of the *N. coli* trophozoites in the feces during the LoSD period in comparison to the HSD period (treatment contrast LoSD vs. HSD: 2.7±0.06 (SE), z = 47.7; p<<0.001). We conclude that our data provide a first indication that starch-rich diet might be responsible for high intensities of infection of *N. coli* in captive individuals and might predispose them for clinically manifested balantidiasis. We discuss the potential nutritional modifications to host diets that can be implemented in part to control *N. coli* infections.

## Introduction

Balantidiasis is a neglected protozoan infection caused by trichostomatid ciliate *Neobalantidium coli* [1] (formerly *Balantidium coli*), inhabiting the hindgut of a variety of mammalian species worldwide [2]. Domestic swine and wild boars are considered as the principle natural reservoirs [3]. In humans, balantidiasis is considered as a zoonotic disease acquired via the fecal-oral route from the reservoir host, usually through contact with swine feces. *N. coli* infection is commonly reported in captive African great apes (prevalence 71%, [4]), but is rarely found in wild apes when examined [4], [5].

The factors affecting the pathogenicity of *N. coli* are unclear. It is considered a harmless commensal in reservoir hosts [2], however, in humans and captive African great apes, especially gorillas, the infections can be manifested by a range of clinical symptoms including chronic diarrhea to bloody dysentery [2], [6], [7], [8], [9]. Moreover, *N. coli* is able to invade other organs in immunocompromised human patients [10], [11], [12], [13] and albeit rare, lethal cases have been reported in humans and captive apes [9], [14], [15], [16].

Although pigs and rats have been used to study *Neobalantidium* infections [17], [18], [19], captive African great apes offer a valuable model, which might help us to unravel the factors contributing to the development of clinical balantidiasis in humans, because both humans and African great apes are not natural hosts for *N. coli* [4] and unlike in pigs and rats clinical outcomes have been observed in apes [2], [6], [7], [8], [9]. The occurrence of *N. coli* in captive African great apes has been explained by (i) the presence of reservoir(s) in captive facilities or/and (ii) differences in the diet of wild and captive apes [4]. It seems that the reservoir hypothesis alone cannot sufficiently clarify the situation as the suid reservoir hosts occur also in the wild [e.g. red river hogs (*Potamochoerus porcus*) being in close contact with free raging apes, Pomajbíková, unpublished data]. On the other hand, it is well-known that the diet of captive great apes significantly differs from that in the wild. Captive ape diets are low in fiber, rich in starch and readily available sugars [20], [21], [22], [23]. Intestinal ciliates from the subclass Trichostomatia are known to possess amylases enabling them to utilize starch, and *in vitro* studies on *N. coli* demonstrated that this ciliate is able to ingest the starch particles [24], [25]. Moreover, a handful of very early experimental studies have suggested a possible effect of a starch-rich diet on *N. coli* infection in pigs, rats and humans [17], [18], [19], [26]. In addition, dietary starch has been shown to enhance ciliate growth in a mutualistic entodiniomorphid ciliate *Troglodytella abrassarti* in captive chimpanzees [27].

Our long-term research on *N. coli* in captive primates [1], [4] provided us with an opportunity to examine the effect dietary starch on the fecal populations of *N. coli* in two groups of captive chimpanzees.

## Materials and Methods

### Ethical statement

This study was authorized by managements of Hodonín and Brno Zoos, the ethics committee of the Institute of Vertebrate Biology; AS CR reviewed and approved the protocol of the study. Our research and the protocol complied with the legal requirements of the Czech Republic (Czech National Council Act No. 246/1992 Coll. the protection of animals against cruelty, amended by Act No. 162/1993 Coll.). Our study was non-invasive. Collection of fecal samples did not include any disturbance of the animals (see the part Sample collection and ciliate quantification). We designed the diets in close collaboration with both Zoos. For welfare and enrichment purposes we paid careful attention to feed chimpanzees variable diets of different food components each day. However, in order to design diets which differ in starch, but stay as much as possible similar in all other nutrients, these diets did not fully meet nutritional requirements of chimpanzees in captivity [21], [28], but we kept specific feeding habits of each chimpanzee group.

### Animals and housing

The study was conducted in the Hodonín Zoo, Czech Republic (chimpanzee group I: adult male Ob, ca. 31 years of age; adult female Zuzana, ca. 37 years of age) from August 12, 2010 to September 19, 2010 and in Brno Zoo, Czech Republic (chimpanzee group II: adult male Fáben, 31 years of age; adult female Nimba, 31 years of age) from November 8, 2010 to December 16, 2010. Chimpanzees in Brno Zoo could utilize three recently repaired indoor enclosures and two outdoor enclosures, while chimpanzees in Hodonín Zoo could use one indoor and one outdoor enclosure. Wooden wool was provided to the chimpanzees for bedding and the indoor enclosures were cleaned once per day by water and disinfectants. Both zoos carry out enrichment programs with focus on feeding enrichment i.e. different ways of hiding, presenting or enriching food provided to animals. Moreover, enclosures are enriched with species specific equipment promoting their natural way of life, including locomotion, resting etc. Chimpanzee had no close contact with humans or other animals kept in the zoos. Our previous study showed that the chimpanzees harbor *N. coli* with no clinical symptoms (e.g. diarrhea) [4].

### Diets and sample collection

During the study, we fed the chimpanzee groups two different diets: a high-starch diet (HSD, average 14.7% of starch) and a low-starch diet (LoSD, average 0.1% of starch). The LoSD was composed only from low starch food items, while the HSD included also potatoes and rice (Table 1). First, we fed chimpanzees the HSD to adapt them to this diet for 7 days, which was followed by a 7-day HSD period with fecal collection. Afterwards there was a 5-day gradual transition to LoSD and another 7-day adaptation LoSD period followed by a 7-day LoSD experimental period when we collected the feces. Throughout the study, daily food intake was 4.502±0.5 kg/animal in wet weight in both zoos. We weighed each dietary item before feeding and, if any of the items remained, after feeding, in order to quantify the exact amount of the total diet ingested.

**Table 1:** Daily intake of experimental diets fed to chimpanzees (means of wet matter, g/day and chimpanzee).

	Hodonín		Brno	
	HSD	LoSD	HSD	LoSD
fruit – both diets^1^	2343±196	2283±245	1085±134	1122±251
vegetable – both diets^2^	1613±228	1739±259	1862±203	1907±225
vegetable/fruit – only LoSD^3^	0	953±108	0	954±171
rice	382±416	0	498±486	0
potatoes (fresh and boiled), rice	618±416	0	428±475	0
dairy products (cottage cheese, yoghurt)	15±42	15±42	92±114	95±118

HSD – high starch diet; LoSD – low starch diet. ^1^Zoo Hodonín: orange, grapes, apple, pineapple, banana, kiwi, pears, watermelon, plum, nectarine; ^1^Zoo Brno: orange, grapes, apple, pineapple, banana, kiwi; ^2^both zoos: pepper, tomato, carrot, kohlrabi, cucumber; ^3^Zoo Hodonín: onion, leek, cauliflower, beet, spring onion, lettuce, Chinese leaves, nectarine; ^3^Zoo Brno: onion, leek, cauliflower, beet, lettuce, Chinese leaves.

We froze the samples of each food item and analyzed them for crude protein, lipid, starch, ash, neutral detergent fiber (NDF), acid detergent fiber (ADF), and acid detergent lignin (ADL), by standard methods approved by the Association of Official Analytical Chemists International [29]. Starch was hydrolyzed with hydrochloric acid and analyzed as free sugars by polarimetry, whereas proteins were determined by using the Carres agents. The resulting values were corrected using optically active compounds dissolved in an ethanol–water mixture [29]. Hemicellulose was calculated as NDF–ADF. Cellulose was calculated as ADF–ADL. LoSD and HSD did not differ in terms protein, fiber and lipid concentrations (Table 2).

**Table 2:** Mean nutritional composition of the low- starch diet (LoSD) and high-starch diet (HSD) consumed by chimpanzees.

	Zoo Hodonín		Zoo Brno	
% of dry matter	HSD	LoSD	HSD	LoSD
NDF	10.9±0.5	13.0±0.9	11.0±0.7	12.7±0.6
ADF	8.8±0.4	10.6±1.0	8.3±0.6	9.7±0.5
ADL	1.5±0.1	1.7±0.2	1.7±0.2	1.7±0.2
Cellulose	7.3±0.4	8.9±0.9	6.6±0.5	8.0±0.5
Hemicellulose	2.1±0.2	2.4±0.2	2.7±0.3	3.0±0.3
Crude protein	11.5±2.1	11.9±1.9	8.5±0.6	8.6±0.6
**Starch**	**18.7±2.0**	**0.1±0.0**	**10.7±1.9**	**0.1±0.1**
Lipids	2.2±0.5	1.9±0.2	1.6±0.3	1.6±0.1
Ash	5.8±0.3	7.0±0.5	5.2±0.3	5.8±0.3

NDF – neutral detergent fiber; ADF – acid detergent fiber; ADL – acid detergent lignin.

### Sample collection and ciliate quantification

In both zoos we collected fecal samples 2–3 times per day from individuals in indoor and outdoor enclosures. We did not pool the samples. The collection of fecal samples was non-invasive and did not cause any disturbance to the animals. We could not assign fecal samples to particular individuals as the ethical considerations prohibited us from separating the animals for the duration of the study.

We immediately placed 5 g of feces in 20 ml of 10% formalin and then transferred the samples to the laboratory of the Department of Pathology and Parasitology of the University of Veterinary and Pharmaceutical Sciences Brno for examination. We quantified the trophozoites of *N. coli* under light microscope (Olympus CX 41) with magnification 400× and by FLOTAC 400 apparatus according to the FLOTAC technique protocol [30] optimized for *Neobalantidium* trophozoite quantification using the FS3 Zinc Sulphate flotation solution (ZnSO_4_. 7H_2_O, s.g. 1.2), [31].

### Statistical analyses

We tested the effect of diet on the number of *N. coli* trophozoites present in feces by a generalized mixed-effects model (GLMM) with Poisson error distribution and log link function. Zoo (Hodonín, Brno) was considered as the experimental unit. The numbers of trophozoites (count data) entered the model as the response variable, while the type of diet was a categorical predictor with two levels (HSD and LoSD) and zoo (Hodonín and Brno) a random effect. The GLMM was fitted in the package lme4 [32], using Laplace approximation for parameter estimations [33]. All statistical analyses were conducted in R 2.13.1 [34].

## Results

**Figure 1:**
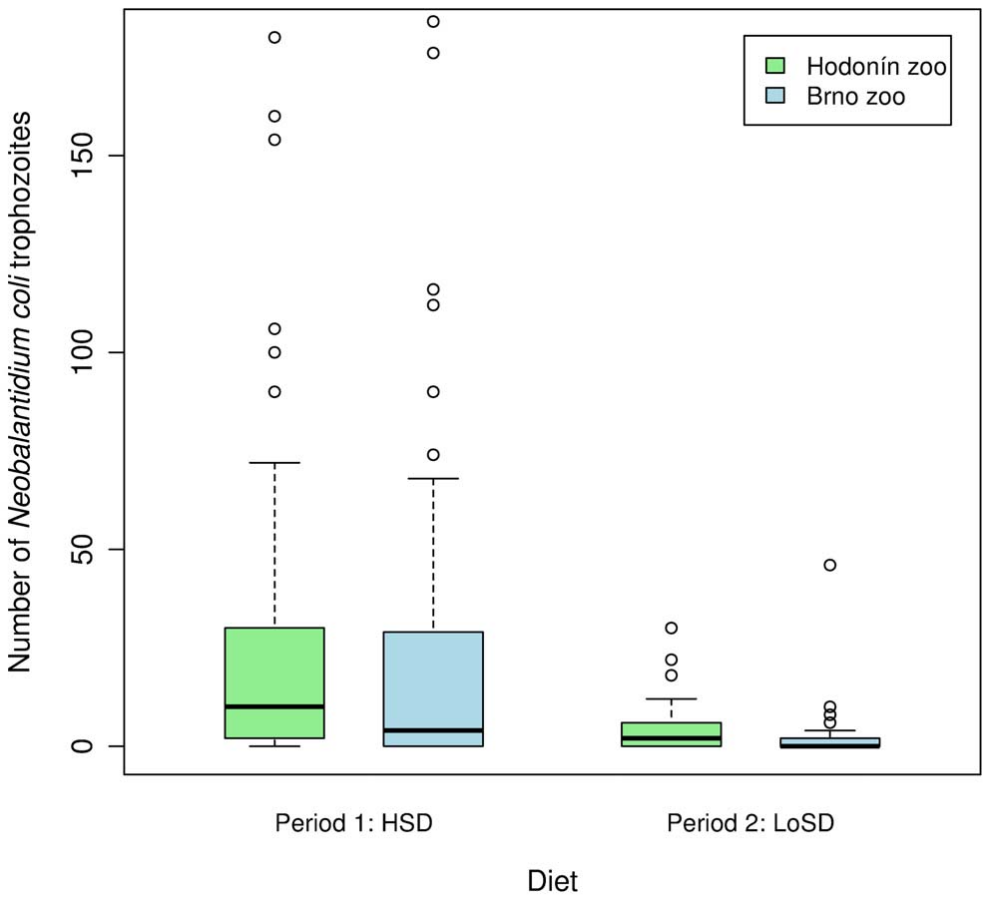
Numbers of *Neobalantidium coli* in chimpanzee feces during low-starch (LoSD) and high-starch (HSD) diets in two study groups.

We examined 58 samples during HSD and 67 samples during LoSD from group I, and 39 samples during HSD and 28 samples during LoSD from group II. We found only trophozoites of *N. coli*; no other parasites were present. No clinical symptoms were observed throughout the whole study period. The numbers of *N. coli* trophozoites were higher during the HSD (mean ± SD: 49.0±134.7) than during the LoSD (3.5±6.8; Fig. 1). The generalized linear mixed-effects model showed a significant difference in the numbers of *N. coli* trophozoites detected in chimpanzee feces between the two diets. The treatment contrast between LoSD and HSD from the GLMM was highly significant (2.7±0.06 (SE), z = 47.7; p<<0.001).

## Discussion

Early studies, carried out nearly a century ago [17], [18], [19], demonstrated that high content of starch in the diet of rats and domestic pigs makes the environment in the caecum and large intestine favorable for multiplying of *N. coli* trophozoites, leading to increased infection intensities. In our model situation, which is much closer to humans, we also recorded lower numbers of *Neobalantidium* trophozoites in feces when chimpanzees were fed with the low starch diet. Although the number of experimental units in our study was low, our results provide an indication that starch-rich diet, which is not typical of wild African great apes [21], [22], [35] might contribute to increased intensities of infection of *N. coli* in captive apes. We point out that a possibility that starch-rich diet might predispose the animals for clinically manifested balantidiasis needs to be further studied.

The assessment of the exact amount of starch passing through the small intestine to the colon of studied chimpanzees is complicated, because resistant starch is determined complexly by several factors and its analytical measurement is difficult [36]. However, studies have shown that the amount of unabsorbed starch entering the colon is directly related to the quantity ingested and that on average 10% of total dietary starch may escape digestion in the small intestine in humans [37], [38], [39]. Salivary amylase protein levels are ∼6–8 times higher in humans than in chimpanzees which suggest that starch digestion in humans is more efficient than that in chimpanzees [40], [41]. Thus it is likely that the proportion of starch entering the colon can be even higher in chimpanzees in comparison to humans.

Clinical balantidiasis in humans and great apes is usually treated by administration of antibiotics, though even repeated treatment does not lead to permanent elimination of *N. coli* in infected apes [7], [15]. Our results imply that low starch diet can maintain low numbers of trophozoites in the intestine. However, as stated above, our experimental diets designed in order to test the impact of dietary starch did not meet fully meet nutritional requirements of chimpanzees in captivity (e.g. in protein and fiber content) [21], [28] and even the LoSD should not be fed to chimpanzees in long-term. Well-balanced diets, more closely resembling the diet of wild great apes should be implemented in accordance with modern zoo standards (see [21], [22], [23], [28]). We also stress that due to low number of experimental units, our results need to be considered as preliminary and more research is necessary to look at long-term effects of different starch levels on the number of trophozoites of *N. coli*. Negative impact of starch-rich (and concurrently low-fiber) diet on various components of intestinal microflora of chimpanzees has been described also by other authors ([42] – increase of *Clostridium perfringens*, [43] – occurrence of trichomonads and mycoplasmas, [27], [44] – increase of *Troglodytella abrassarti*).

Development of effective antiparasitic strategies requires substantial knowledge of host-parasite interactions, including direct effect of host nutrition on the parasites. To date, most of the research has focused on utilization of nutritional alternations in the control of helminthiases parasitizing livestock [45], [46], [47]. Taking into consideration the results of diet experiments with *Neobalantidium* infection in rats and domestic pigs [17], [18], [19], together with results of our study on chimpanzees, we call for more studies supporting the indications that low starch diet can be implemented as supportive treatment of balantidiasis in addition to antibiotics. In addition, Green and Scully [26] cured four cases of balantidiasis in human patients by putting the patients on milk diet with reduction or complete elimination of starches, while there was no uniformity in dietary management of balantidiasis in other two studies, which noted diet only incidentally [48], [49]. Maybe it is time to reappraise the old studies, describing the impact of diet on *N. coli* infections and future studies should survey the possibilities of nutritional adjustments as a part of parasite control strategies not only in livestock farming, but also in zoo husbandry and potentially in human medicine.
